# Cry Protein Crystals: A Novel Platform for Protein Delivery

**DOI:** 10.1371/journal.pone.0127669

**Published:** 2015-06-01

**Authors:** Manoj S. Nair, Marianne M. Lee, Astrid Bonnegarde-Bernard, Julie A. Wallace, Donald H. Dean, Michael C. Ostrowski, Richard W. Burry, Prosper N. Boyaka, Michael K. Chan

**Affiliations:** 1 Department of Chemistry and Biochemistry, The Ohio State University, Columbus, OH, United States of America; 2 Department of Veterinary Biosciences, The Ohio State University, Columbus, OH, United States of America; 3 Department of Molecular and Cellular Biology, The Ohio State University, Columbus, OH, United States of America; 4 Tumor Microenvironment Program, The Comprehensive Cancer Center, The Ohio State University, Columbus, OH, United States of America; 5 Department of Neuroscience, The Ohio State University, Columbus, OH, United States of America; UMASS Medical School, UNITED STATES

## Abstract

Protein delivery platforms are important tools in the development of novel protein therapeutics and biotechnologies. We have developed a new class of protein delivery agent based on sub-micrometer-sized Cry3Aa protein crystals that naturally form within the bacterium *Bacillus thuringiensis*. We demonstrate that fusion of the *cry3Aa *gene to that of various reporter proteins allows for the facile production of Cry3Aa fusion protein crystals for use in subsequent applications. These Cry3Aa fusion protein crystals are efficiently taken up and retained by macrophages and other cell lines *in vitro*, and can be delivered to mice *in vivo* via multiple modes of administration. Oral delivery of Cry3Aa fusion protein crystals to C57BL/6 mice leads to their uptake by MHC class II cells, including macrophages in the Peyer’s patches, supporting the notion that the Cry3Aa framework can be used to stabilize cargo protein against degradation for delivery to gastrointestinal lymphoid tissues.

## Introduction

Protein delivery has emerged as a safe and powerful tool to deliver a wide array of therapeutic candidates to cells and tissues [1]. This has resulted in its use in several medical applications including but not limited to vaccination [2, 3], regenerative medicine [4, 5], cancer therapeutics [6] and imaging [2, 7]. One of the major challenges in the development of protein-based therapies is getting the protein therapeutic to the cellular target. Protein delivery platforms that can both protect the protein therapeutic during delivery, and facilitate their uptake by the target cells have a role to play in this arena. Liposomes [1, 8], polymeric beads [7, 9], spores [10–12] and virus particles [13, 14] have been explored extensively for this purpose, but their production costs and modest protein loads are potential limitations. As such, alternative platforms with favorable properties that overcome these limitations are of considerable interest.

Herein, we describe a new class of protein delivery agent that is easy to produce and isolate, is efficiently uptaken into cells, and protects its cargo (protein) from proteolytic degradation. This platform is based on sub-micrometer-sized protein crystals that naturally form within the bacterium *Bacillus thuringiensis* (*Bt*) [15–17]. The crystals are comprised of crystal-forming Cry proteins that have found widespread use as biopesticides and in genetically-modified (GM) crops. Given that these GM crops are currently used in food production, Cry proteins are generally considered to be safe for ingestion by humans [18, 19].

**Fig 1:**
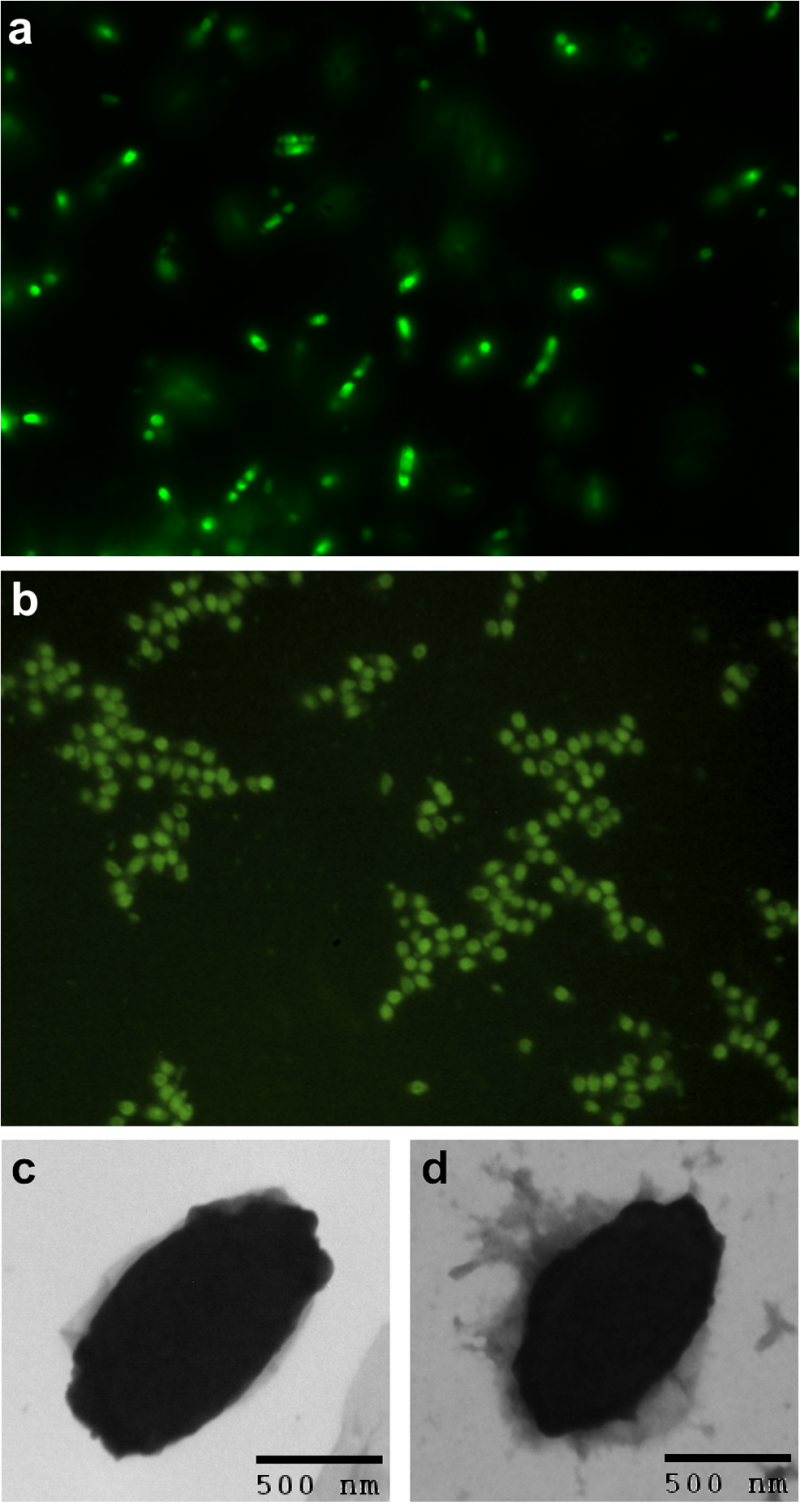
Production of Cry3Aa-GFP crystals. Fluorescence images of Cry3Aa-GFP crystals (a) expressed in *B*. *thuringiensis* cells after 48 h growth, and (b) as purified from cells. Electron micrographs of purified (c) Cry3Aa protein crystals, and (d) Cry3Aa-GFP fusion protein crystals.

Cry proteins have been stably fused to other toxins and enzymes in the past using genetic engineering. These include galactose-binding domain of the nontoxic ricin B chain (BtRB) to Cry1Ac [20], and the replacement of domain III of Cry1Ac with garlic agglutinin [21], However, most if not all of these modifications were focused on enhancing insecticidal activity. As part of structural studies focused on obtaining the structure of *Bt* Cry3Aa in bicelles [22, 23], we explored the generation of its fusion to GFP and mCherry as a means to distinguish its crystals from those of detergent and lipid. As expected, overexpression of the resultant Cry3Aa-GFP and Cry3Aa-mCherry fusion proteins in *Bt* resulted in the bacteria being fluorescent (Fig 1A and S1 Fig). Surprisingly, the addition of the reporter domain did not block crystal formation. Rather, the fusion proteins still formed crystals within the *Bt* cells.

Given this finding and the known resistance of Cry3Aa protein crystals to degradation by proteases at pH ≤ 8 at room temperature [24], we hypothesized that the Cry3Aa crystals could potentially serve as a general platform for encapsulating proteins for various applications. Some of the attractive features of this system would include the ease of producing Cry3Aa crystals in high amounts in acrystalliferous strains of *Bt*, and the regular ordering of the proteins in the crystalline framework [24]. Further support for the highly ordered nature of Cry3Aa crystals in *Bt* is provided by the recent work of Sawaya *et al*. [25] who were able to determine the structure of Cry3Aa at 2.9 Å resolution by streaming whole cells bearing these crystals through an X-ray free electron laser beam.

Due to their sub-micrometer size, we surmised that Cry3Aa fusion protein crystals might be efficiently taken up by mammalian cells, making them potentially useful as a protein delivery vehicle. To test this hypothesis, we have performed *in vitro* and *in vivo* protein delivery studies with crystals of Cry3Aa fused to various reporter proteins. Our cellular studies show that these crystals are efficiently taken up by macrophages and other cell lines, while the animal studies demonstrate their effective delivery to mice via multiple modes of administration. Both the *in vitro* studies with different cell lines and *in vivo* mouse studies suggest that the Cry3Aa framework stabilizes its cargo protein against degradation, suggesting their potential as a delivery agent for a variety of protein therapeutics.

## Methods

### Bacterial strains and plasmids

Plasmid pHT315 and the gene for expressing Cry3Aa crystal proteins were obtained from the *Bacillus* Genetic Stock Center (BGSC) at The Ohio State University (OSU). Transformation of the vector was done into *E*.*coli* strains XL10 (Stratagene). Competent bacteria (*Bacillus thuringiensis* strain *Bt*407) were generated from stocks supplied by BGSC using methods described previously [26].

To create the Cry3Aa expression system, the pHT315 vector [26] was mutated to engineer AfeI and XhoI restriction sites upstream of the *cry3A* gene site. The Cry3Aa promoter with STAB-SD sequence [17] was amplified from *Bacillus thuringiensis* var. *tenebrionis* and subcloned between the AfeI and XhoI sites. The *cry3Aa* gene was then cloned into the XhoI and BamHI sites of the vector using the In-Fusion HD Cloning Kit (Clontech Inc) producing vector pHT315-*cry3Aa*.

Plasmids containing the mCherry or GFP coding sequence were kindly provided by Dr. Berl Oakley (OSU) and Ms. Cynthia Hatfield (OSU), respectively, while the *luciferase* gene was obtained from the commercial vector, pGL4 Basic (Promega). These reporter genes were each amplified and inserted in frame at the 3’ end of *cry3Aa* in pHT315-*cry3Aa* using BamHI and KpnI restriction sites. All clones obtained were verified using DNA sequencing at the OSU Plant Microbe Genomics Facility.

### Production and purification of Cry3Aa fusion protein crystals

The expression of Cry3Aa and each Cry3Aa fusion protein was carried out with *Bt407* cells transformed with the appropriate plasmid, in a modified Schaefer’s Sporulation Medium (SSM) [27] bearing sporulation salts. *Bt407* cells were provided to the BGSC by Dr. Didier Lereclus [26]. Cells were grown at 25°C for 72 h with vigorous aeration after which, the crystal-spore mixture was harvested using centrifugation at 8000 rpm for 10 min in an Avanti J25 ultracentrifuge (Beckman Coulter). The pellet was washed with sterile distilled water and purified using continuous density gradient of 60% solution of iodixanol (Optiprep; Sigma Aldrich) in a SW28 swinging bucket rotor in a Beckman L7 ultracentrifuge. One band containing ~80% crystals (under phase contrast microscope) was extracted from the iodixanol gradient and washed with autoclaved water and 50 mM sodium acetate pH 5.0 for 5–10 times to remove all iodixanol solution. In cases where the extracted crystals were not deemed sufficiently pure based on manual inspection by microscope, the crystals were further purified by binding to a CM-cellulose column (50mM sodium acetate pH 5.0), followed by washing with 10 mM phosphate (pH 6.8), and then eluting the crystals from the column using Tris-EDTA (pH 8.0) buffer [28].

Cells and crystals were examined in 50% glycerol by phase contrast and fluorescent microscopy during sporulation growth and during crystal purification to verify the presence of fluorescent crystals and to monitor their purification. Protein concentrations from crystals were estimated for both the crystalline forms and the solubilized forms using the Bradford assay (Biorad) since it has been determined to be the most reliable method [29] and is commonly used in the field [30, 31].

### Transmission electron microscopy of Cry3Aa crystals

Transmission electron microscopy (TEM) imaging was performed at the OSU Campus Microscopy and Imaging Facility (OSU CMIF). Samples were prepared by dispersing 0.5 mg Cry3Aa and Cry3Aa-GFP crystals in 1 mL cell culture water (Lonza). The samples were sonicated for 5 min and were stained with 6 drops of 3% phosphotungstic acid. The carbon grids were prepared by slowly dipping an Ultrathin Carbon Type-A 400 Mesh Copper Grid (Ted Pella) into solutions of either the Cry3Aa or Cry3Aa-GFP protein crystal three times and drying the grid at ambient temperature. A FEI Tecnai G2 transmission electron microscope operating at 200 kV was used to obtain TEM micrographs of the crystals.

### Cry3Aa fusion protein crystal uptake into macrophages

The murine macrophage-like cell line RAW264.7 (ATCC Number: TIB-71) was seeded at 5 x 10^4^ cells/well on an 8-well chamber slide and incubated overnight at 37°C in 5% CO_2_. Cells were then washed with 1X phosphate-buffered saline (PBS) to remove non-adherent cells, followed by incubation with 120 μg/mL of Cry3Aa-GFP crystals in 200 μL of Dulbecco's modified Eagle's medium (DMEM) supplemented with 10% fetal bovine serum (FBS) and penicillin/streptomycin (P/S) for 15 min to 4 h at 37°C in 5% CO_2_. At the end of each incubation period, cells were washed three times with PBS to remove any free Cry3Aa-GFP crystals, and fixed with 4% paraformaldehyde in PBS for 20 min at room temperature. The fixed cells were washed three times with PBS and counterstained with 4,6-diamidino-2-phenylindole (DAPI). Cells were washed extensively with PBS and coverslipped using Gel/Mount. Images were obtained using an Axioscope 40 microscope equipped with an Axiocam HRc camera (Zeiss).

To confirm that Cry3Aa protein crystals facilitate GFP uptake, RAW264.7 macrophages were seeded at 5 x 10^4^ cells/well on an 8-well glass bottom chamber slide (ibidi) and incubated overnight at 37°C in 5% CO_2_. Cells were then washed with PBS to remove non-adherent cells, followed by incubation with 2 μM of either Cry3Aa-GFP crystals or GFP protein in 200 μL DMEM supplemented with 10% FBS and P/S for 1 h to 4 h at 37°C in 5% CO_2_. At the end of the 1-h incubation period, the media from individual wells were transferred to the corresponding wells of an empty 8-well chamber slide. Cells were washed with PBS once, followed twice by 20U/mL heparin in PBS to remove any surface-bound Cry3Aa-GFP crystals or GFP protein. Images were captured using a Nikon Eclipse Ti epifluorescence microscope. The media set aside prior to imaging were then transferred back to their corresponding wells containing the experimental cells. Cells were further incubated for another 3 h at 37°C in 5% CO_2_. At the end of the 4-h time point, cells were stained with 0.2 μg/mL Hoechst 33342 (Life Technologies), and washed once with PBS, followed twice with 20U/mL heparin in PBS. Images were captured using a Nikon Eclipse Ti epifluorescence microscope.

Confocal studies were carried out at the OSU CMIF. RAW264.7 cells were incubated with 120 μg/mL of Cry3Aa-mCherry crystals in 200 μL DMEM supplemented with 10% FBS and P/S for 12 h at 37°C in 5% CO_2_. Cell seeding, washing, incubation, and fixing were performed in a similar fashion described above.

### Electron microscopy of macrophages

RAW 264.6 cells were plated at 1.5 x 10^5^ cells/well on a 4-well poly-L-lysine-coated chamber slides and incubated overnight at 37°C in 5% CO_2._ Prior to incubation with Cry3Aa-mCherry crystals, cells were washed twice in PBS and were then incubated in 400 μL of DMEM containing 25 μL of 120 μg/mL Cry3Aa-mCherry crystals for 2 h at 37°C in 5% CO_2_. At the end of the incubation period, cells were washed 4 times with PBS to remove any free Cry3Aa-mCherry crystals. 500 μL of fresh DMEM with no Cry3Aa-mCherry crystals were added to the washed cells for an additional 2 h incubation at 37°C in 5% CO_2_. Cells were washed with PBS three times prior to fixation with gluteraldehyde for EM study.

#### Cellular lifetime analysis of the Cry3Aa-mCherry crystals

Bone marrow-derived macrophages (BMMs) were obtained from C57BL/6J female mice and prepared by flushing bone marrow from femurs of the mice, and cultured in DMEM containing 50 ng/mL CSF-1 for 3 days in non-treated tissue culture dishes. BMMs were seeded at 5 x 10^4^ cells/well on an 8-well chamber slide and incubated overnight at 37°C in 5% CO_2_. Cells were washed in 1 mL 1X PBS and were then incubated with 15 μL of 120 μg/mL Cry3Aa-mCherry crystals in 200 μL DMEM for 1 h at 37°C in 5% CO_2_. After 1 h incubation, cells were washed 3 times with 1 mL PBS to remove any free Cry3Aa-mCherry crystals, and 200 μL fresh DMEM were added to the cells. Cells were then incubated at 37°C in 5% CO_2_ for the designated incubation period (96-, 72-, 48-, 36-, 24-, 12-, 8-, 4-, 2-h) to allow for internalization of the Cry3Aa-mCherry crystals. At the end of incubation, cells were fixed and stained following the protocols described above.

To quantitate the mCherry fluorescence in the BMMs, approximately 50 images of the cells at each incubation period were collected with an X20 objective. More than 70+ randomly selected cells were traced and analyzed for each time point using the Metamorph image analysis software (Universal Imaging).

### Cellular retention analysis of Cry3Aa-GFP protein crystal in macrophages

To prepare for the Texas Red dextran-labeled cells, 5 x 10^4^ RAW264.7 macrophages were incubated with [10 mg/mL] Texas Red dextran (Invitrogen) in 600 μL DMEM for 18 h at 37°C in 5% CO_2_. The Cry3Aa-GFP-labeled cells were prepared by seeding 5 x 10^4^ RAW264.7 macrophages on an 8-well chamber slide and incubated with Cry3Aa-GFP crystals for 2.5 h at 37°C in 5% CO_2_. At the end of incubation, the cells were washed with PBS three times to remove excess labeling reagent. The Texas Red dextran RAW cells were then added to the Cry3Aa-GFP RAW cells and incubated for 2 h. At the end of the 2-h incubation period, cells were washed with PBS three times and fixed with 4% paraformaldehyde in PBS for 20 min at room temperature. The fixed cells were washed three times with PBS before counterstained with DAPI. Cells were washed extensively with PBS and coverslipped using Gel/Mount. For each well, multiple fluorescence images were sequentially taken using an Axioscope 40X microscope in a grid-like fashion to cover the entire cellular population. These images were then manually inspected to tabulate the number of cells containing either Texas Red dextran only, Cry3Aa-GFP crystals only, or both particles.

### Cry3Aa-mCherry crystals uptake by primary mouse fibroblast cells

Primary mammary mouse fibroblasts were prepared following the protocol described by Trimboli *et al*. [32]. The cells were then incubated with 15 μL of 0.6 μg/mL Cry3Aa-mCherry crystals in 200 μL of DMEM for 1.5 h at 37°C in 5% CO_2_. At the end of the incubation, cells were washed with PBS three times to get rid of any free Cry3Aa-mCherry crystals. 200 μL of DMEM were then added to the washed cells for a further 1.5 h incubation before fixing with paraformaldehyde.

### Animals and Diet

C57BL/6 and C57BL/6 albino mice were housed in a temperature-controlled (20–22°C) room on a 12 h-light/dark cycle in an animal facility maintained by the University Laboratory Animal Resources, The Ohio State University, Columbus, OH. All protocols were approved by the Institutional Animal Care and Use Committee at The Ohio State University (Protocol # 2008A0210) and conducted in accordance with the Office of Animal Health Welfare (OLAW) Public Health Service Policy Guide for the Care and Use of Laboratory Animals. Mice were maintained on a standard chow diet and allowed ad libitum access to water and food.

### Luminescence imaging of Cry3Aa-luciferase crystals in mice

The protein concentrations of Cry3Aa-luciferase crystal and recombinant Firefly luciferase protein (Creative Biomart) were determined using the Bradford protein assay (BioRad). Measurement of the *in vivo* luminescence was performed on an IVIS 100 system (Xenogen). C57BL/6 albino mice were anaesthetized with Ketamine/Xylazine at a concentration of 10 mg/kg of body weight of mouse for imaging. D-luciferin was provided at the concentration of 125 mg/kg of body weight in PBS (15 mg/mL stock). For testing the viability of Cry3Aa-luciferase crystal delivery for the different routes of administration, a 4.85 mg/mL stock solution of Cry3Aa-luciferase crystals was used. 100 μL of Cry3Aa-luciferase stock crystal solution was given to mice by either intraperitoneal injection or oral gavage, while 25 μL was delivered for nasal route.

### Activity and lifetime measurement of Cry3Aa-luciferase crystals

For evaluation of the oral stability and lifetime of Cry3Aa-luciferase crystals, C57BL/6 albino test mouse was gavaged with 100 μL of Cry3Aa-luciferase crystal stock solution followed by D-luciferin (Caliper Life Sciences) at a dose of 125 mg/kg of body weight of mice. For comparison, a second mouse was gavaged with 100 μL of 25 mg/mL luciferase protein followed by D-luciferin. For both experiments, additional D-luciferin was provided at 20 min intervals by oral gavage to maintain the luminescence intensity. Similar comparisons were done for intraperitoneal and nasal routes except that 25 μL volumes were used through nasal route.

### Flow cytometry analysis of Cry3Aa-GFP crystals in gut-associated lymphoid tissues

Peyer’s patches, mesenteric lymph nodes and spleen were collected after 8 or 12 h of oral intubation of 1 mg of Cry3Aa-GFP crystals from five C57BL/6 mice and processed to obtain single cell suspension. Naïve mice or mice provided with GFP protein were used as controls. Cells were washed, resuspended in staining buffer (PBS, 1%BSA, 0.01% NaN_3_) and labeled by incubating for 30 min at 4°C with a combination of the fluorescent anti-mouse antibodies, namely anti-CD11b and anti-IAb (BD Biosciences). Cells were then washed twice in staining buffer and analyzed by flow cytometry on a C6 Flow cytometer (Accuri Cytometers). Sample groups were experimentally analyzed on the C6 Flow cytometer in triplicate and statistical analyses using the paired t-test were performed.

## Results

### Production and characterization of Cry3Aa fusion protein crystals

The production of intact Cry3Aa fusion protein crystals was confirmed by their isolation using density gradient ultracentrifugation. Both the Cry3Aa-GFP and Cry3Aa-mCherry crystals were found to contain full-length fusion proteins upon SDS-PAGE electrophoresis of solubilized crystals (S1 Fig) with a two-band pattern similar to that observed for Cry3Aa when expressed in *Bt* [33] or recombinantly in *E*.*coli* [34]. Isolated crystals were strongly fluorescent (Fig 1B and S1 Fig) demonstrating the proper folding of the mCherry and GFP reporter proteins within the Cry3Aa crystal lattice. This fluorescence could be maintained for several weeks.

Electron microscopy performed on native Cry3Aa crystals (Fig 1C **)** and Cry3Aa-GFP (Fig 1D **)** crystals showed them to be of similar size, approximately 0.5 microns in each dimension. We subsequently demonstrated that this same protocol could be used to produce other Cry3Aa fusion protein crystals (e.g. Cry3Aa-luciferase) with retention of protein function.

### Cry3Aa fusion protein crystal uptake by macrophages

**Fig 2:**
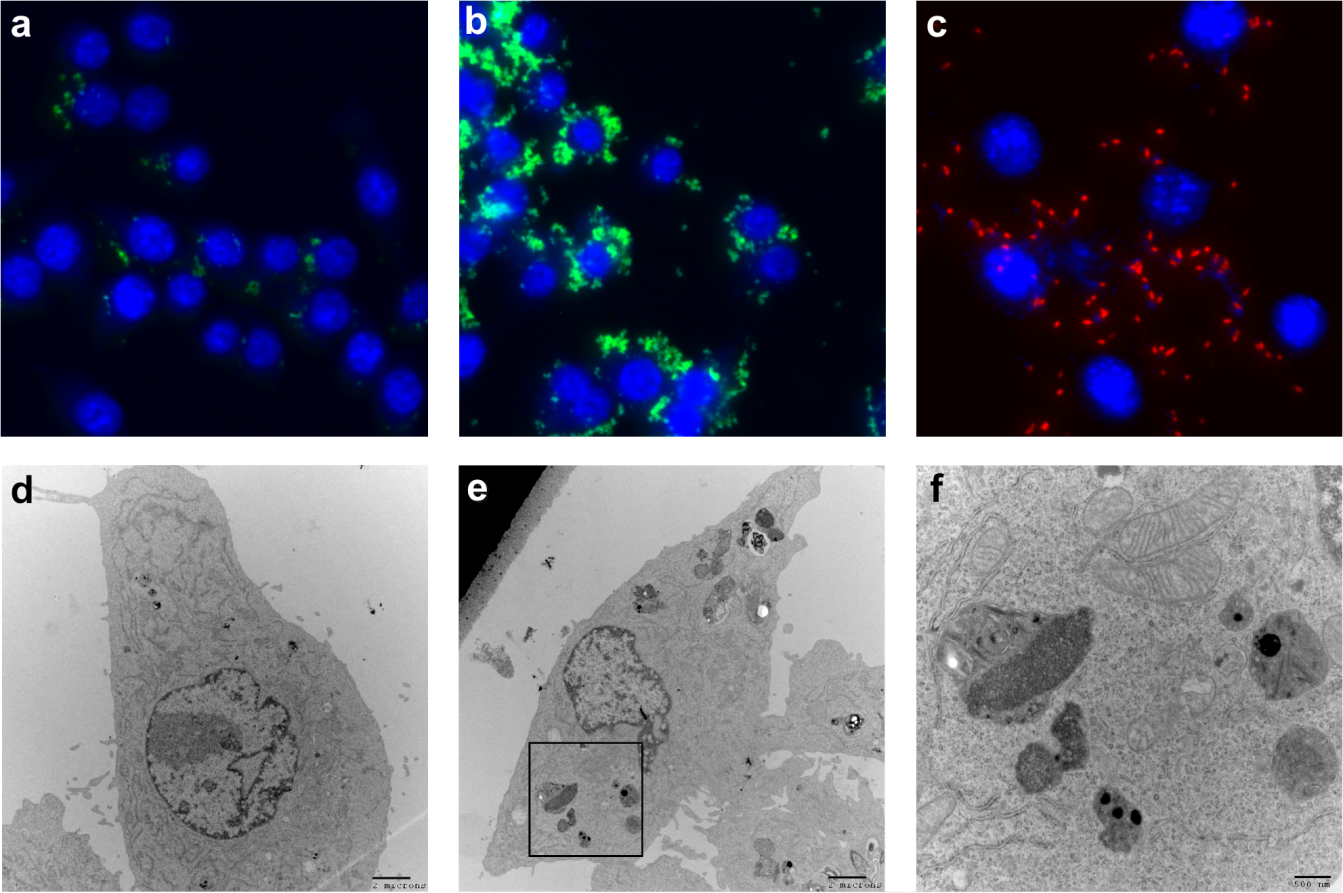
Fluorescence and TEM images of Cry3Aa-GFP and Cry3Aa-mCherry crystals uptaken into cells. Macrophage cells (RAW264.7) were incubated with Cry3Aa-GFP crystals for different time periods. Fluorescence images at (a) 15-min, demonstrating fast initial uptake and at (b) 1-h, where the green fluorescence intensity reached its maximum value. Note the punctate fluorescence pattern, which supports uptake of the whole crystal. (c) Red fluorescence image of primary mouse fibroblasts after 1.5 h incubation with Cry3Aa-mCherry protein crystals is not as intense as seen with macrophages. TEM images of (d) untreated RAW264.7 cells, and (e) cells treated with Cry3Aa-GFP. The Cry3Aa-GFP treated cells exhibit a distinct cytoplasmic particulate not observed in the control cells. (f) Enlarged TEM image of the cytoplasmic particulate observed in the Cry3Aa-GFP treated macrophages highlighting the likely crystal degradation in the cytoplasm.

Given that nanoparticles and microparticles have found application as protein and small molecule delivery vehicles, the delivery of Cry3Aa protein crystals to macrophages by normal cellular uptake mechanisms was investigated. RAW264.7 macrophages were incubated with the Cry3Aa-GFP crystals over a period of 4 h and the uptake and cellular location of these crystals were examined using epifluorescence microscopy. As indicated in Fig 2, Cry3Aa-GFP crystals were readily taken up by the macrophages as early as 15 min (Fig 2A) with maximal internalization after 1 h (Fig 2B). There was no significant increase in fluorescence signal with longer incubation. Incubation of RAW264.7 cells with GFP protein showed no significant uptake after 1 h, or even 4 h, based on the absence of a measurable fluorescence signal (not shown).

To determine the localization of the Cry3Aa fusion protein crystals in cells, confocal images of RAW264.7 macrophages after 12 h incubation with the Cry3Aa-mCherry crystals were collected (S2 Fig). Analysis of the confocal images indicated that the crystals were localized in the cytoplasm of the cell, an observation further supported by TEM images of cells treated with Cry3Aa-GFP crystals (Fig 2D–2F). It is important to note that the only crystals seen in the TEM were intracellular and no crystals were seen attached to the outside membrane of cells.

One factor that influences the possible applications of Cry3Aa fusion protein crystals is their intracellular stability and that of their cargo proteins. While most proteins undergo rapid proteolysis in cells and thus have a short cellular lifetime (for GFP in LA-9 cells, the half-life is 26 h [35]), we hypothesized that encapsulated proteins within the Cry3Aa fusion protein crystal framework might be shielded and therefore less susceptible to such rapid degradation. To test this hypothesis, BMMs were pulse-labeled with Cry3Aa-mCherry crystal-containing media for 1 h, and then chased with Cry3Aa-mCherry-crystal-free media over a period of 96 h. These studies showed that even after 96 h, the Cry3Aa-mCherry-treated cells still retained 30% of the maximal fluorescence observed at 12 h (S3 Fig), thus supporting the ability of Cry3Aa fusion protein crystals to protect and enhance the cellular half-life of their cargo protein.

Cellular retention is another important property that impacts the potential utility of Cry3Aa fusion protein crystals. For example, given the strong fluorescence of the Cry3Aa-GFP and Cry3Aa-mCherry protein crystals and their stability in cells, one potential application of this protein delivery technology is cellular tagging for *in vivo* and *in vitro* studies [36]. However, certain microparticles have been observed to be prone to rapid release by eukaryotic cells, including lymphocytes and monocytes, which would complicate their application for this purpose [37]. To evaluate whether Cry3Aa fusion protein crystals were subjected to facile cellular release events, two distinct groups of RAW264.7 macrophages were prepared. One group was tagged with Texas Red dextran and a second group was tagged with Cry3Aa-GFP crystals. After washing each group to remove excess labels, the two groups of macrophages were mixed and incubated for 2 h. The effect of mixing the two populations of macrophages was then examined by epifluoresence microscopy. The resulting fluorescence images showed that at most only a few cells (< 5%) contained both Cry3Aa-GFP crystals and Texas Red dextran (S4 Fig), suggesting that if there is any cellular release of the Cry-3Aa-GFP crystals, it is minimal.

### Cry3Aa fusion protein crystal uptake by primary mouse fibroblasts

Since macrophages are naturally phagocytic, the potential for Cry3Aa fusion protein crystals to promote uptake in other cell types was also evaluated. Primary mouse fibroblasts were incubated with Cry3Aa-mCherry crystals for 1.5 h, and their cellular internalization was assessed using epifluoresence microscopy. Cry3Aa-mCherry crystals were taken up by the fibroblast cells (Fig 2C), suggesting that uptake of crystals is possible by multiple types of cells.

### Cry3Aa fusion protein crystal delivery to mice

**Fig 3:**
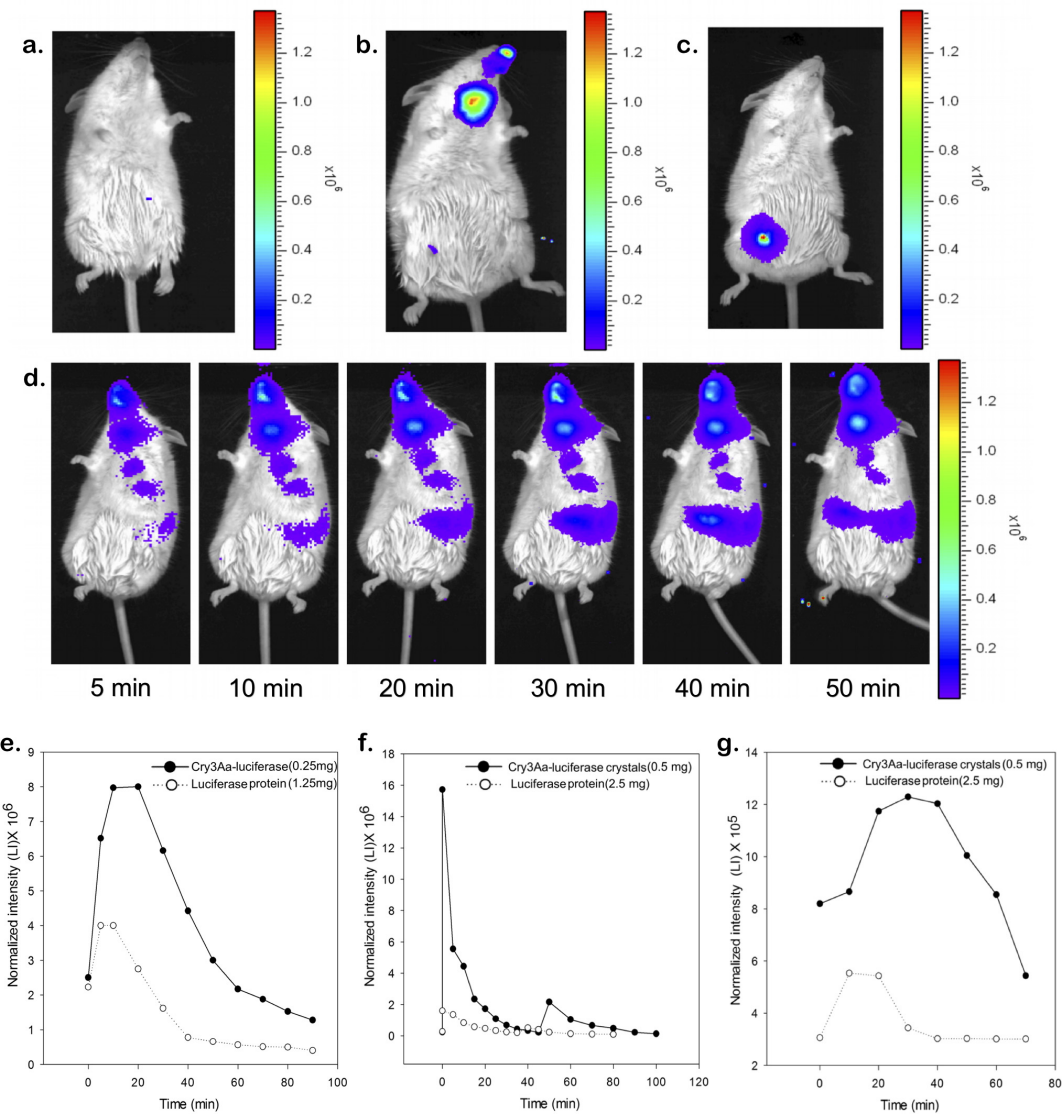
Bioluminescence of Cry3Aa-luciferase crystals delivered to mice. (a) No Cry3Aa-luciferase crystal control (b) nasal uptake, and (c) intraperitoneal injection. The luminescence measurements were taken 10 min after crystal and D-luciferin delivery. (d) Luciferase activity following oral gavage of Cry3Aa-luciferase crystals after 5, 10, 20, 30, 40, and 50 min. The D-luciferin substrate was replenished at 20 min intervals. Activity and lifetime of luciferase activity of Cry3Aa-luciferase crystals (0.5 mg or 0.25 mg as indicated in the figure) and luciferase protein (2.5 mg or 1.25 mg as indicated in the figure) delivered to C57BL/6 mice via (e) nasal spray, (f) intraperitoneal injection and (g) oral gavage. Measurements were made by selecting a region of interest (ROI) in the area where the maximal intensity was obtained from the crystal and the protein. Lifetime of luminescence was measured in the selected ROI over the indicated times. D-luciferin was replenished every 20 min. The lifetime of the luciferase activity of the Cry3Aa-luciferase crystals was found to be higher that of the recombinant luciferase protein in each route of delivery, supporting the ability of the Cry3Aa crystal framework to protect the luciferase protein from degradation.

In addition to its ability to facilitate the uptake of proteins into cells, the Cry3Aa fusion protein crystal platform was also explored as a tool to aid in the *in vivo* delivery of protein therapeutics to humans and animals. Since imaging deep tissues in animals with fluorophores like GFP and mCherry is hindered by hemoglobin and water absorption [38], we switched to using luciferase as the reporter of choice given their excellent compatibility with mammalian models, including mice and other rodents [39]. Cry3Aa-luciferase crystals were prepared, and the luciferase activity of these crystals was verified *in vitro*. Cry3Aa-luciferase crystals were then delivered to C57BL/6 albino mice by different modes of administration, namely intraperitoneal injection, nasal uptake, and oral gavage. As shown in Fig 3, non-invasive *in vivo* imaging of treated mice indicates successful delivery of Cry3Aa-luciferase crystals by all three routes of administration.

For delivery of therapeutics, the oral route is preferred because it is the least invasive [40–42]. It also provides one of the most efficient routes for inducing immune response. The long mucosal lining (one of the largest areas in the body) exposes the cells of the immune system to foreign antigens, while the secretory antibodies already present in the mucosa lead to rapid activation to known agents [43, 44].

One of the major challenges to the delivery of therapeutics by the oral route is the harsh acidic and protease-rich environment of the midgut. This can lead to degradation of the therapeutic particularly in the case of proteins. Notably, it had been previously shown that native Cry3Aa crystals degrade slowly at low pH—even in the presence of trypsin [45], and thus we hypothesized that Cry3Aa fusion protein crystals might be able to survive these harsh conditions within the human midgut, and in so doing, serve as a shell to protect the protein cargo from denaturation and proteolytic degradation. As such, we decided to test the stability of the Cry3Aa crystals and their efficacy of absorption in the gut cavity.

Cry3Aa-luciferase crystals and purified luciferase protein were delivered by oral gavage to mice and the luciferase activity was monitored over time. While the activity of the orally delivered luciferase protein disappeared within 30 min, that of the Cry3Aa-luciferase crystals could be observed in the mouse for more than 1 h (Fig 3G), thus allowing for tracking of Cry3Aa fusion protein crystal migration from the mouth to the gut (Fig 3D). This retention of luciferase activity by the Cry3Aa-luciferase crystals in the mouse gut is significant as it supports the notion that Cry3Aa fusion protein crystals have stabilizing properties that could be used to aid in the oral delivery of protein therapeutics.

Another major challenge for oral delivery of therapeutics is particle absorption. It has been shown that unlike for soluble proteins, uptake of particulate agents such as virus-like particles, liposomes or whole bacteria require the presence of organized lymphoid follicles (e.g. Peyer’s patches) in the intestine [44, 46, 47]. Research on particle absorption into the oral cavity has been shown to be very specific to the nature of the particles, their *in vivo* stability, their interactions with epithelial tissues in the mucosa of the intestine, and their downstream destination and stability [43]. Common mechanisms that can lead to transfer across the epithelial barrier of the mucosa include endocytosis by specific M-cells present in the epithelial lining of Peyer’s patches, or transport between enterocytes in the intestine itself. In the case of antigens, proteins delivered across the epithelial barrier of either mechanism would be delivered to the gut-associated lymphoid tissues (GALT) for downstream processing.

To evaluate the uptake efficiency of orally delivered Cry3Aa-GFP crystals and their fate in the intestinal mucosa, 1 mg of Cry3Aa-GFP crystals was delivered to C57BL/6 mice via oral intubation. Control mice were provided with either GFP protein or no protein. After 8–12 hours post feeding, lymphoid follicles in the intestine, namely, the Peyer’s patches and the mesenteric lymph node were collected. Spleen from each mouse was also isolated. The tissues were processed and labeled with either anti-IAb antibody labeled with phycoerythrin (PE) fluorophore suitable for detection of MHC class II molecule bearing cells or with anti-CD11b antibody linked to an allophycocyanin (APC) fluorophore that would specifically label those cells that were macrophages. The use of both these antibodies would allow for detection of any antigen-presenting cells in these tissues that harbor the GFP-bearing antigen using flow cytometry. The final percentages were calculated from at least three separate rounds of mouse experiments.

**Fig 4:**
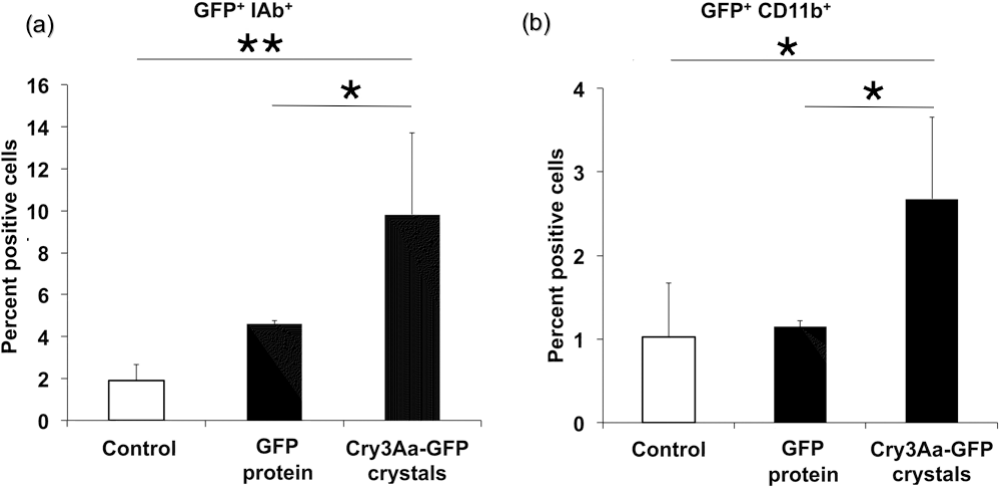
Uptake of Cry3Aa-GFP crystals by antigen presenting cells in Peyer’s patches. (a) Percentage of IAb^+^ cells (MHC-class II) in Peyer’s patches that retained GFP after 8 hours post oral intubation of Cry3Aa-GFP crystals or GFP protein (GFP^+^IAb^+^). (b) Percentage of CD11b^+^ cells (macrophages) in Peyer’s patches that retained GFP after 8 hours post oral intubation of Cry3Aa-GFP crystals or GFP protein (GFP^+^CD11b^+^). Data are represented as mean ± std. dev of samples assayed; * (*P<0*.*05*); ** (*P<0*.*01*). The flow cytometry analyis of GFP^+^ live cells was made with an Accuri C6 flow cytometer after immunostaining of cell surface markers with fluorescent antibodies.

Flow cytometric measurements clearly showed that after 12 hours, GFP-positive cells were present only in the Peyer’s patches (S5 Fig) but not in the mesenteric lymph nodes or spleen cells (not shown), suggesting a pathway for migration through the lymphatics in a manner similar to other known particulate delivery agents (i.e. from Peyer’s patches to mesenteric lymph node to spleen) [43, 44, 48]. Cry3Aa-GFP^+^ cells were also positive for IAb (6.8%) and CD11b (2.7%) markers indicating the involvement of macrophages and other MHC Class II antigen-presenting cells in taking up the crystals (Fig 4). By comparison, Peyer’s patches of naïve mice (controls) and mice receiving GFP protein showed a much lower percentage of GFP^+^CD11b^+^ cells (1.0% and 1.1%, respectively), indicating an improved uptake of GFP when associated with Cry3Aa crystals. Given that CD11b^+^ cells account for less than 12% of myeloid cells in murine Peyer’s patches [49], the Cry3Aa-GFP crystals are able to stimulate myeloid cells at least 10% more than the controls. Since protective immune response in mice requires less than 0.1% of antigen-specific T-cells [50, 51], these crystals should be of extremely high potential if an antigen fused Cry3Aa crystals can be taken up with similar efficacy.

## Discussion

The cellular and animal studies described herein affirm the potential of Cry3Aa fusion protein crystals as a novel protein delivery platform. While a number of different nanoparticle and microparticle systems have been explored for this purpose, notable features of Cry3Aa fusion protein crystals include their simplicity of purification and ability to accommodate a variety of different fusion protein partners. These features make this platform potentially one of the cheapest, and yet, most robust systems to encapsulate a protein cargo within a sub-micrometer size particle. Most nano- or microparticle-based approaches involve separate synthesis of the particle and the cargo protein, followed by an additional step to encapsulate the protein within the particle [1, 14, 52–57]. For the Cry3Aa fusion protein crystal platform, all these steps are consolidated into a single step—greatly reducing production complexity and costs.

One significant advantage that the Cry3Aa fusion protein crystal platform has over existing nano- or micro-particle approaches is the high protein load. For a typical polymeric bead, the cargo protein makes up only a small fraction of the particle [58], with the rest of the particle being the polymeric framework. In contrast, the Cry3Aa fusion protein crystal platform has a very high protein cargo density since each protein cargo molecule is directly fused to a Cry3Aa crystal-forming protein. As each protein crystal is comprised of ~10^5^–10^6^ Cry3Aa protein molecules, a similar amount of cargo protein is encapsulated. This feature imparts the Cry fusion protein crystal platform with potentially one of the highest concentrations of cargo protein available in a nano- or micro-particle framework.

Another appealing feature of the crystal framework is the protection afforded to the cargo protein. Based on the relative Cry-luciferase crystal and luciferase protein lifetimes in the gut, and the differential uptake of Cry-GFP and GFP protein by the Peyer’s patches, it appears that the crystal can play a significant role in aiding the target payload reach its destination (cells or tissues). Presumably, the crystal framework helps its protein cargo survive the harsh acidic environment of the gut.

Given these properties, one possible application of Cry fusion protein crystals could be the delivery of antigens to the Peyer’s patches and other regions of the gut-associated lymphoid tissues for further antigen processing. Since the GFP signal from the flow analysis is mainly observed in antigen presenting cells, especially macrophages, we speculate that the route by which the crystals reach the Peyer’s patches and the further downstream processing could be similar to one of the routes which bacteria or other particulate matters use to reach the lymphoid tissues of the intestine [43, 48, 59–61]. However, determination of their exact route will require further experimentation.

In summary, we have developed a novel framework for delivering proteins to both mammalian cells and animals, utilizing a naturally-forming Cry3Aa protein crystal that is cheap and easy to produce. These desirable properties together with its flexibility to accommodate different proteins and high cargo protein content make the Cry3Aa fusion protein crystal platform a promising candidate for use in a wide variety of enzyme-based therapies and biotechnological applications. Future studies will include the elucidation of the mechanism of uptake of Cry fusion protein crystals and the exploration and development of new uses for this novel protein delivery platform.

## Supporting Information

S1 Fig Fluorescence images and SDS-PAGE gel of Cry3Aa fusion protein crystals. (a) Cry3Aa-mCherry crystals expressed in *B*.*thuringiensis* cells after 48 h growth, and (b) as purified from cells. (c) Purified crystals of Cry3Aa-GFP (lane 1) and Cry3Aa-mCherry (lane 2) were solubilized for 3 h using 50 mM sodium carbonate (pH 10.5) solution and the purity of the intact full-length complex was confirmed by SDS-PAGE. Lane 3 shows molecular weight marker (MW).
(TIF)

S2 Fig Overlay of confocal and phase-contrast images of Cry3Aa-mCherry crystals in a macrophage. RAW264.7 cells were incubated with Cry3Aa-mCherry crystals for 12 h before fixing with paraformaldehyde and staining with DAPI. Red fluorescence was observed inside the cytoplasm of the cell.
(TIF)

S3 Fig Stability of Cry3Aa-mCherry crystals chased in primary mouse macrophages. Cells were treated with Cry3Aa-mCherry crystals for 1 h, and then chased with crystal-free medium over a period of 96 h. The cellular stability of the Cry3Aa-mCherry crystals is supported by the long lifetime of the fluorescence signal. Notably, after 96 h in crystal-free media, the fluorescence intensity remains at 30% of its maximum value (12-h incubation). The control (Ctrl) refers to non-treated cells at the 96-h time point.
(TIF)

S4 Fig Probing cellular retention of Cry3Aa-GFP protein crystals. RAW264.7 macrophages were separately incubated with either (a) Cry3Aa-GFP crystals, or (b) Texas Red dextran. The two samples were washed with DMEM medium, mixed, and incubated for 2 h. (c) The clear separation of the Cry3Aa-GFP and Texas Red fluorescence into different cells supports the notion that once taken up, there is minimal release of the Cry3Aa-GFP crystals by cells.
(TIF)

S5 Fig Flow cytometry samples showing uptake of Cry3Aa-GFP crystals by Peyer’s patches. 1 mg of Cry3Aa-GFP was provided to C57BL/6 mice by oral intubation and Peyer’s patches isolated after 12 h were processed to obtain a single cell suspension. Naïve mice provided with PBS served as controls. Cells were incubated with either anti-IAb antibody bearing PE fluorophore (panels a,b,e) or anti-CD11b antibody bearing APC fluorophore (panels c,d,f) and analyzed on Accuri C6 flow cytometer. Figure panels represent a single run of each sample indicative of a significant increase in uptake of Cry3Aa-GFP crystals by Peyer’s patches in comparison to GFP protein. The final percentages were obtained from a set of at least three such experiments. The paired t-test was used to determine statistical significance.
(TIF)
