# Supplementary figures and images for: Cry Protein Crystals: A Novel Platform for Protein Delivery
Source: PLoS One. 2015 Jun 1;10(6):e0127669. doi: 10.1371/journal.pone.0127669 (PMC4451076; doi:10.1371/journal.pone.0127669)

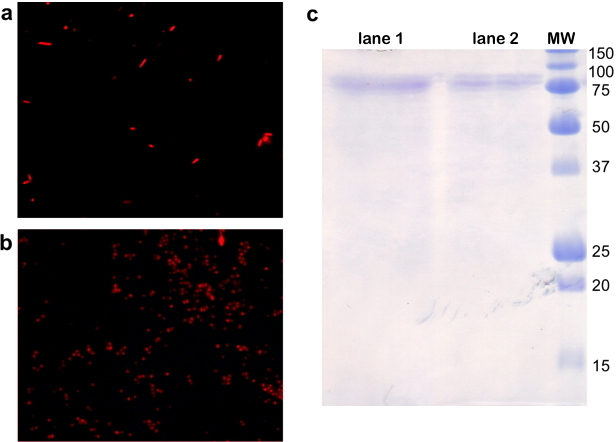

Supplement: S1 Fig — (a) Cry3Aa-mCherry crystals expressed in B.thuringiensis cells after 48 h growth, and (b) as purified from cells. (c) Purified crystals of Cry3Aa-GFP (lane 1) and Cry3Aa-mCherry (lane 2) were solubilized for 3 h using 50 mM sodium carbonate (pH 10.5) solution and the purity of the intact full-length complex was confirmed by SDS-PAGE. Lane 3 shows molecular weight marker (MW). (TIF) [file pone.0127669.s001.tif]

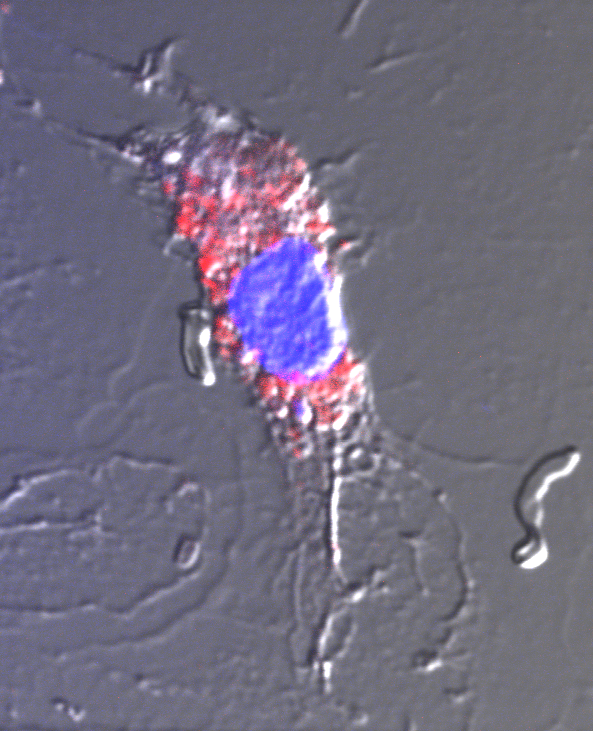

Supplement: S2 Fig — RAW264.7 cells were incubated with Cry3Aa-mCherry crystals for 12 h before fixing with paraformaldehyde and staining with DAPI. Red fluorescence was observed inside the cytoplasm of the cell. (TIF) [file pone.0127669.s002.tif]

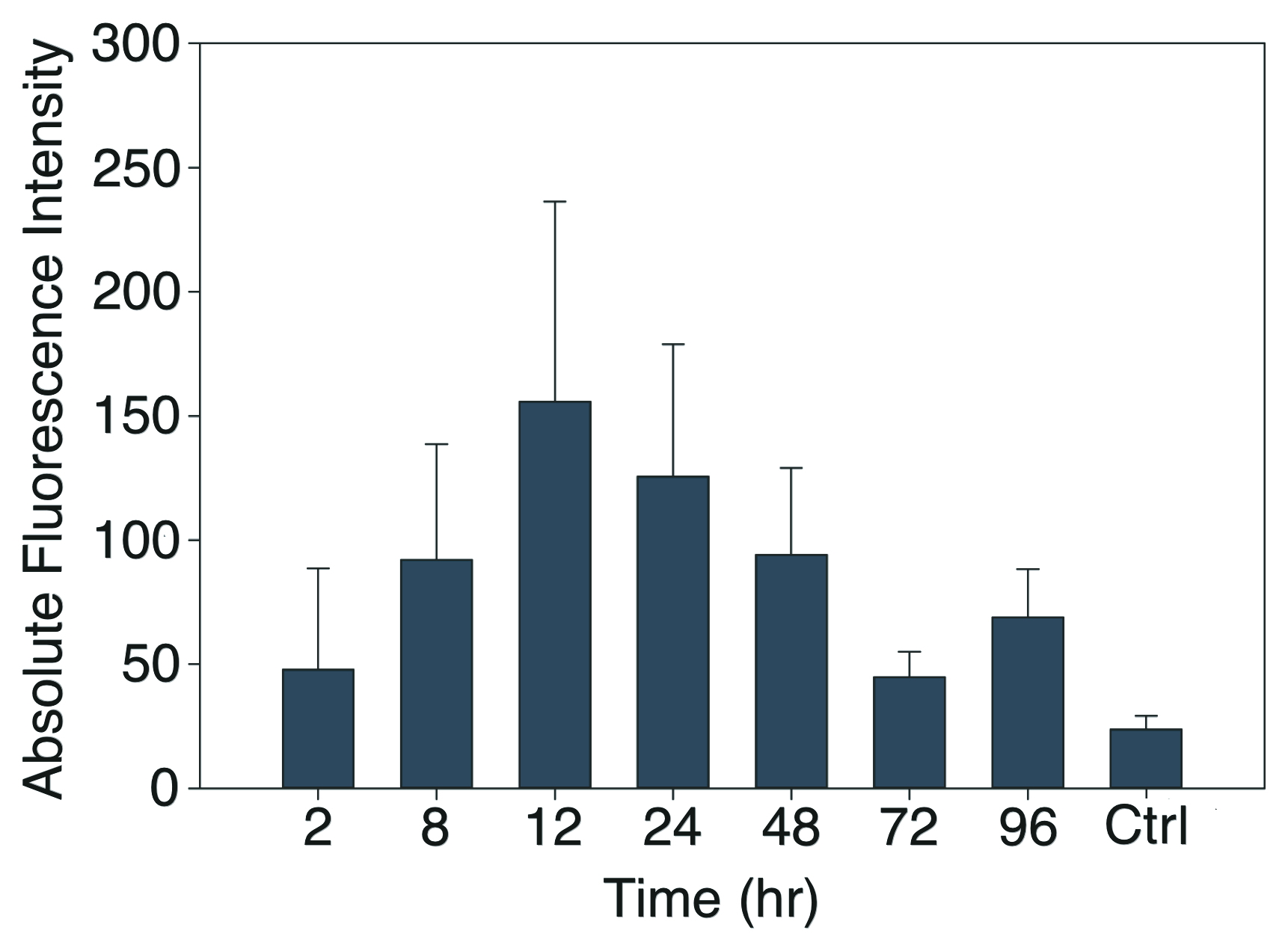

Supplement: S3 Fig — Cells were treated with Cry3Aa-mCherry crystals for 1 h, and then chased with crystal-free medium over a period of 96 h. The cellular stability of the Cry3Aa-mCherry crystals is supported by the long lifetime of the fluorescence signal. Notably, after 96 h in crystal-free media, the fluorescence intensity remains at 30% of its maximum value (12-h incubation). The control (Ctrl) refers to non-treated cells at the 96-h time point. (TIF) [file pone.0127669.s003.tif]

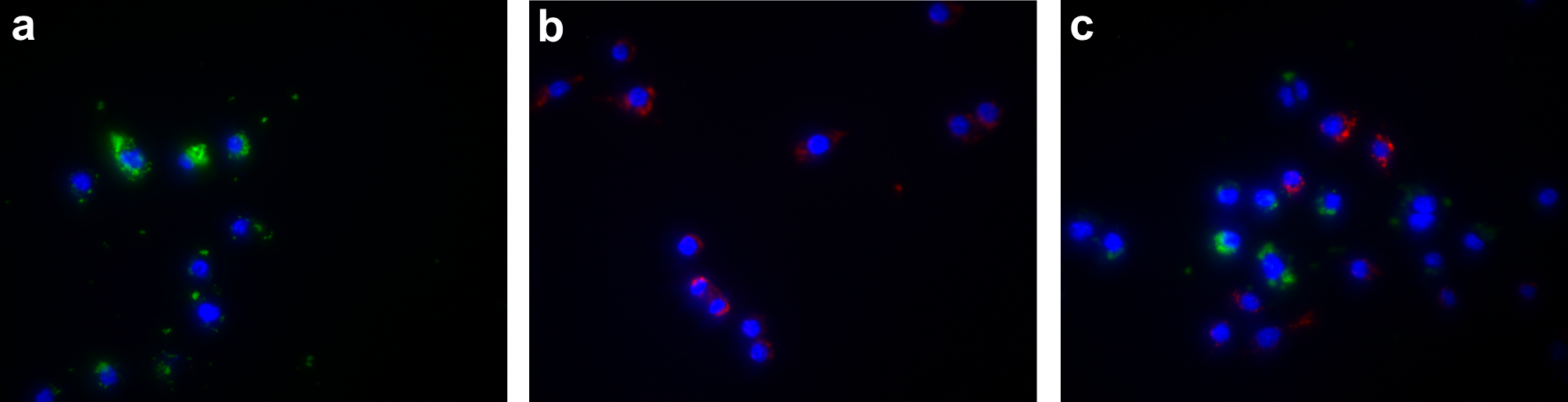

Supplement: S4 Fig — RAW264.7 macrophages were separately incubated with either (a) Cry3Aa-GFP crystals, or (b) Texas Red dextran. The two samples were washed with DMEM medium, mixed, and incubated for 2 h. (c) The clear separation of the Cry3Aa-GFP and Texas Red fluorescence into different cells supports the notion that once taken up, there is minimal release of the Cry3Aa-GFP crystals by cells. (TIF) [file pone.0127669.s004.tif]

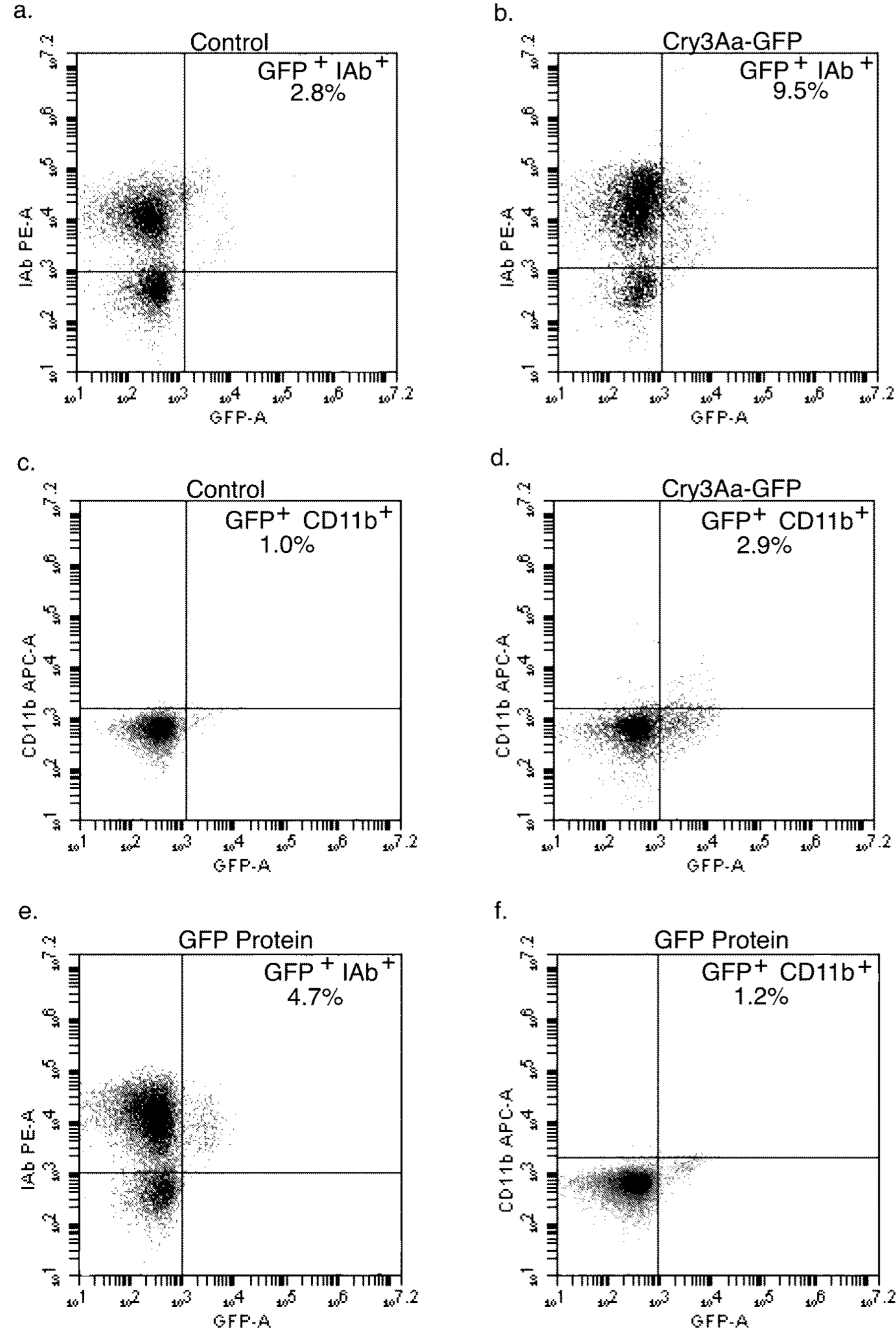

Supplement: S5 Fig — 1 mg of Cry3Aa-GFP was provided to C57BL/6 mice by oral intubation and Peyer’s patches isolated after 12 h were processed to obtain a single cell suspension. Naïve mice provided with PBS served as controls. Cells were incubated with either anti-IAb antibody bearing PE fluorophore (panels a,b,e) or anti-CD11b antibody bearing APC fluorophore (panels c,d,f) and analyzed on Accuri C6 flow cytometer. Figure panels represent a single run of each sample indicative of a significant increase in uptake of Cry3Aa-GFP crystals by Peyer’s patches in comparison to GFP protein. The final percentages were obtained from a set of at least three such experiments. The paired t-test was used to determine statistical significance. (TIF) [file pone.0127669.s005.tif]
